# Considerations for the development and implementation of electronic immunization registries in Africa

**DOI:** 10.11604/pamj.2018.30.81.11951

**Published:** 2018-05-29

**Authors:** Apophia Namageyo-Funa, Anita Samuel, Peter Bloland, Adam Macneil

**Affiliations:** 1 Global Immunization Division, Center for Global Health, Centers for Disease Control and Prevention

**Keywords:** Electronic immunization registries, immunization, information system, registry

## Abstract

While paper-based immunization registries are the prevalent form of documenting individual-level immunization service delivery in Africa, some countries are interested in transitioning to electronic immunization registries (EIRs) which have the potential to transform immunization data into useable information for decision making to optimize the performance of immunization programs. This report discusses opportunities and challenges in the adoption of EIRs in the African continent.

## Commentary

Traditionally, immunization data at the health facility level have been collected and managed using paper-based tools, including registry books, tally sheets, and summary reports, which are not readily analyzed and accessible to provide input for program decisions. However, in recent years there has been an explosion of information and communications technology (ICT) [1] that presents new opportunities for collection, management and use of immunization data at both the individual and aggregate level. While ICT terminology may differ, depending on the user and context, for clarity we have provided a list of definitions for this report (Table 1). At the most basic level, immunization registries (IRs) are systems that collect and report individual-level vaccine administration record data and can be paper-based (PBIR) or electronic (EIRs). Limited data are available on the experiences of establishing and developing EIRs in Africa [2, 3]. The stage of development and implementation of EIRs varies by country and most activities are at early stages of implementation [2, 4]. While some countries have EIRs that are stand alone, others have IRs that are integrated as immunization modules within other parts of an electronic medical records system within a facility [5]. This comment article discusses opportunities and challenges in the adoption of EIRs in the African continent (Table 2). EIRs have the potential to facilitate the collection and analysis of individual-level vaccine administration data, which can transform this data into usable information to support programmatic decision making for improving the quality of immunization service delivery [6]. In particular, EIRs have the potential to facilitate data exchange and sharing within and across systems and stakeholders, which offers opportunities at the point of service delivery to help parents and healthcare workers track children to ensure they receive all needed vaccines and opportunities for staff managing district, regional and national immunization programs to identify populations at high risk for vaccine preventable diseases to target interventions and resources efficiently and effectively. However, there are also many challenges associated with implementing EIRs. Knowing and understanding the challenges helps inform the successful development and implementation of EIRs.

**Table 1:** Common terms used to discuss Electronic Immunization Registries

Clinical Decision Support (CDS) is the feedback provided to a healthcare worker through an IR or IIS that provides clinical follow up tools, job aids, or other decision point support.
eHealth Strategy is a strategic plan that incorporates the overall aim of a variety of systems (e.g. IR, IIS, HMIS) in order to meet national programmatic goals.
Functional Standards are a description of the type, variety, and amount of data that will be collected through a system and how it would be shared with other systems. This includes minimal data sets, a data dictionary that describes the data elements and how they are entered into the system, methods of how data would be shared with other systems, and describes the functions of the system.
Information and Communications Technology is an umbrella term used to encompass all rapidly emerging, evolving and converging computer, software, networking, telecommunications, Internet, programming and information systems technologies.
Health Management Information System (HMIS) or Health Information System (HIS) is a system that collects and stores multiple programs aggregate data in order to show progress on status towards programmatic goals through indicators.
Immunization Registry (IR) is a system that collects and reports individual level immunization record data. Used primarily by health workers.
Immunization Information System (IIS) includes an aggregate data collection system that can be linked with an immunization registry. Used for public health reporting.
Service Level Agreement (SLA) is a contract between the software provider and the user that describes how the system will be maintained over a defined period of time. This includes maintenance, bug fixes, upgrades, as well as troubleshooting or help desk support.
System is a paper or electronic based method of collecting, storing, and using information. A system is not only the technological component, but also the workforce that collects, reports, and uses the data.
System Integration is the combining of two systems into one through a mapping process of identifying common data elements, such as a unique identifier. This allows for data sets to be combined and for additional analyses to be run, while maintaining separate systems. Functional standards provide the basis for how systems are linked or made “interoperable”.
System Requirements are the translation of the public health programmatic needs, described in the functional standards and user requirements documentation, into the technical specifications that an IT company uses to create the software and develop the system.
User Requirements are a stratified description of the functions the end user, at a variety of levels, would need the system to do. The user requirements ensure that the workflows of the users, whether clinical or public health or at the district or national level, are met. This is also where visualizations, such as dashboards, would be created in order to meet data use needs by the end user to achieve programmatic goals.

**Table 2:** Opportunities and challenges of implementing Electronic Immunization Registries

Opportunities
Providing capability to track child vaccination records across multiple sources
Combining vaccine administration information from different sources into a single child vaccination record
Reminding families when an immunization is due or has been missed
Helping providers and parents determine when vaccinations are due to ensure children get the vaccinations they need
Simplifying and improving immunization data reporting to and from health facilities
Identifying populations at high risk for vaccine-preventable diseases to target interventions and resources efficiently and effectively
Linking to other information systems (e.g., Civil Registration and Vital Statistics, newborn metabolic screening) to create an electronic birth record, which could improve estimates of target populations, tracking of other child health interventions, and sustainability
**Challenges**
Increased burden of data collection by health workers
Limited electricity and poor internet/telecommunication connectivity
Lack of plans for interoperability among multiple EIR pilots
Lack of governance or steering mechanism with stakeholders for a successful system implementation
Lack of long-term funding for EIR maintenance and system upgrades
Lack of ability to provide necessary, continuous training of workforce and helpdesk functions

**Awareness and reaching a common understanding**: To be successful, moving from a PBIR to an EIR requires knowledge acquisition and sharing to occur among stakeholders at different levels in the health system, including representatives from clinical care, public health and information technology (IT) sectors. The technical language and terminology used when discussing development and implementation of EIRs might not be understood in the same way by experts from the different sectors. For example, the way in which a term such as “interoperability” is conceptualized or understood within the public health and clinical care sectors might be quite different than the way it is conceptualized or understood within the IT sector. Clear definitions of terms to ensure a common understanding of the technical language used is critical to developing and implementing an EIR. The development team should be well acquainted with the experiences of other countries [2-4], including challenges, successes and any identified missed opportunities that might inform the development team about what does or does not work and why as they plan the design, development and implementation of an EIR.

**Considerations on timing and process**: There is no “one size fits all” approach, as every country has a unique context in which its immunization information system operates. However, there are important national level questions that need to be addressed and programmatic activities that should occur prior to the adoption of an EIR. For instance: does an immunization registry already exist and is it effective? Is it the appropriate time to move to an electronic system (e.g. availability of IT infrastructure, human and financial resources to support implementation)? What are the pros and cons of a PBIR vs an EIR? What are the pros and cons of a standalone vs an integrated system? What is the process for development of the system requirements? An initial step is ensure that the overall immunization information system is well described and documented from health facility to national level, including gaps and future needs. As health workers at the health facility would be the first level users of an EIR, understanding how their workflow operates would minimize the potential for additional burden of reporting. During the initial phase of implementation, a hybrid approach to EIR adoption might be used, with EIRs adopted in settings, such as in urban health centers, while keeping continuity of the PBIR everywhere else. Ensuring the support and ownership of the development and implementation process by the Ministry of Health should be considered as well as aligning efforts with overall country health information system needs.

**Functional standards**: With the technology advancements in ICT, numerous options exist for countries to develop, adopt, and implement EIRs. No global consensus exists around functional standards that define the answers to such questions as: what is the minimum set of data elements that are required for an effectively functional EIR? What is the minimum set of functional capabilities that an effective EIR needs to have? What are the required data sharing standards (e.g. information exchange, archiving information, protecting privacy)? A globally accepted set of functional standards would help guide system development, whether commercial products or “home-grown” systems, while better ensuring data quality, analysis, and ability to transmit and share information between information systems and with stakeholders. Evidence of country specific standards has been observed in the United States where the American Immunization Registry Association has worked with diverse partners to support registry development, implementation and evaluation [7].

**Information system requirements**: Information system requirements are a set of instructions to the developer that clearly and specifically describe what the information system needs to be able to do to perform as intended by the users and relevant stakeholders. Articulation of precise information system requirements that are agreed to and clearly understood by all relevant parties is a critical step in moving from PBIRs to EIRs. The requirements also ensure that the final product meets the needs of the national immunization program and Ministry of Health, and adheres to any globally accepted functional standards. It is important to ensure that a Service Level Agreement is created with the vendor or system developer in order to ensure that the information system requirements can be met. Information system upgrades might be necessary when a new vaccine is introduced or additional data elements are desired. However, before adding new data elements, a review of data currently collected must occur to determine if it can be used to achieve programmatic goals.

**Integration with other information systems**: While an EIR predominantly contains patient-level vaccine administration data, it also serves as a source for aggregation to create synergies with other information systems. In particular, linkage of EIRs with Civil Registration and Vital Systems or other information systems focused on maternal and child health (e.g., newborn metabolic screening programs) could be used to create electronic birth registries which could improve estimates of target populations and tracking of other child health interventions [8]. Linking of EIR with other information systems could also enhance cost-effectiveness and sustainability. Immunization-related modules have been included in some patient medical record systems not focused specifically on immunizations [5]. These modules, however, were often add-ons not developed specifically to meet the needs of the immunization stakeholder users. As a result, such modules have been infrequently used by health facility staff or used in parallel to existing paper-based systems, thus increasing the burden of data collection on facility staff. Reasons for not using the electronic system or using both electronic and paper systems vary but might include lack of or unreliable electricity, lack of time, or lack of available staff. Integration of immunization modules within an existing electronic medical record system can serve to facilitate the transition from a PBIR to an EIR and the use of the data by relevant stakeholders if developed carefully. To effectively link and sustain EIRs with other information systems, protocols must be in place on how data are shared and protected. Approaches on data sharing and protection that have been widely used include data sharing agreements, security protocols and levels of user access of the system for privacy [9]. As patient-level data are inherently vulnerable, security layers will need to be added to the data that is commonly used public health aggregated data. Also the efficient use of existing hardware and infrastructure can be leveraged.

## Conclusion

While PBIRs are the prevalent form of documenting immunization service delivery, there is an interest among countries in transitioning from PBIRs to EIRs. The availability of EIRs could offer many advantages for data use in program planning and decision making. Transitioning from PBIRs to EIRs will improve the work flow and decrease the disruptions to the delivery of immunization services. Even with this aim in mind, some countries use both paper-based and electronic systems in parallel [5]. Countries within Africa are at different stages in the development and implementation of EIRs. The applicability of the opportunities and challenges described above may vary based on the country context and stage of implementation-some countries are having initial discussions on the topic, some are piloting EIRs, while others are exploring building on existing electronic medical record systems. As more countries explore and share their experiences with developing and implementing EIRs, collection of evidence from Africa and the lessons learned will guide other countries in Africa and elsewhere on transitioning from PBIRs.

## Competing interests

The authors declare no competing interests.
